# Supplementary material for: Integrative analysis of high-dimensional RCT and RWD subject to censoring and hidden confounding
Source: Lifetime Data Anal. Author manuscript; Available in PMC 2025 Aug 4. (PMC12317910; doi:10.1007/s10985-025-09654-1)
Supplement: supp [file NIHMS2081880-supplement-supp.pdf]

Supplementary to "Integrative Analysis of High-dimensional RCT and RWD Subject to Censoring and Hidden Confounding"

Xin Ye[1,4], Shu Yang[2]*, Xiaofei Wang[3], Yanyan Liu[4]

[1] *School of Statistics and Mathematics, Guangdong University of Finance and Economics*

[2] *Department of Statistics, North Carolina State University*

[3] *Department of Biostatistics and Bioinformatics, Duke University*

[4] *School of Mathematics and Statistics, Wuhan University.*

Appendix A: technical details for Section 3

Firstly, we rewrite the regression model with more concise notations. Let $\mathbf{Y}_i = \log(\widetilde{T}_i) - \mu(\mathbf{Z}_i)$, $\widehat{\mathbf{Y}}_i = \log(\widetilde{T}_i) - \widehat{\mu}(\mathbf{Z}_i)$, $\mathbf{Y} = (\mathbf{Y}_i, i = 1, 2, ..., n)$, and $\widehat{\mathbf{Y}} = \left(\widehat{\mathbf{Y}}_i, i = 1, 2, ..., n\right)$. Denote that

$$\Lambda = \text{diag}\left\{A_i - e(\mathbf{Z}_i), i = 1, 2, ..., n\right\}, \quad \widehat{\Lambda} = \text{diag}\left\{A_i - \widehat{e}(\mathbf{Z}_i), i = 1, 2, ..., n\right\}.$$

Let $\rho(\boldsymbol{\theta}) = \sum_{j=1}^{p} \rho(|\theta_j|; \lambda_1) + \rho(\|\boldsymbol{\theta}_{\mathcal{G}}\|; \lambda_2)$. Then the loss function (4) can be written into

$$\ell_{\lambda_1,\lambda_2}(\boldsymbol{\theta}, \widehat{\eta}|\mathcal{OB}) = \left\|\mathbf{W}^{1/2}\left(\widehat{\mathbf{Y}} - \widehat{\Lambda}\mathbf{U}\boldsymbol{\theta}\right)\right\|_2^2 + \rho(\boldsymbol{\theta}).$$

Proof of Theorem 1

The proof follows the lines of Fan and Peng (2004). We aim to show that for any given ϵ, there is a large enough constant M such that, for large n, we have

$$\Pr\left\{\min_{||\Delta\boldsymbol{\theta}||_2 = M} \ell_{\lambda_1,\lambda_2}(\boldsymbol{\theta}^* + R_n\Delta\boldsymbol{\theta}, \widehat{\eta}|\mathcal{OB}) > \ell_{\lambda_1,\lambda_2}(\boldsymbol{\theta}^*, \widehat{\eta}|\mathcal{OB})\right\} \geq 1 - \epsilon,$$

*Corresponding author: syang24@ncsu.edu

where $\Delta\boldsymbol{\theta} \in \mathbb{R}^{2p}$ is an increment vector. This implies that with probability converging to 1, there is a local minimum $\widehat{\boldsymbol{\theta}}$ such that $\|\widehat{\boldsymbol{\theta}} - \boldsymbol{\theta}^*\|_2 = O_p(1)R_n$. It can be seen that

$$\begin{aligned}
&\ell_{\lambda_1,\lambda_2}(\boldsymbol{\theta}^* + R_n\Delta\boldsymbol{\theta}, \widehat{\eta}|\mathcal{OB}) - \ell_{\lambda_1,\lambda_2}(\boldsymbol{\theta}^*, \widehat{\eta}|\mathcal{OB}) \\
&= \left\|\mathbf{W}^{1/2}\widehat{\Lambda}\mathbf{U}\Delta\boldsymbol{\theta}\right\|_2^2 R_n^2 + 2\Delta\boldsymbol{\theta}\mathbf{U}^{\mathrm{T}}\widehat{\Lambda}\mathbf{W}\left(\widehat{\Lambda}\mathbf{U}\boldsymbol{\theta}^* - \widehat{\mathbf{Y}}\right)R_n + \{\rho(\boldsymbol{\theta}^* + R_n\Delta\boldsymbol{\theta}) - \rho(\boldsymbol{\theta}^*)\} \\
&= (\mathrm{I}) + (\mathrm{II}) + (\mathrm{III}).
\end{aligned}$$

We first deal with (I). It can be deduced that

$$\begin{aligned}
(\mathrm{I}) &= R_n^2\left\|\mathbf{W}^{1/2}\Lambda\mathbf{U}\Delta\boldsymbol{\theta}\right\|_2^2 + R_n^2\left\|\mathbf{W}^{1/2}(\Lambda - \widehat{\Lambda})\mathbf{U}\Delta\boldsymbol{\theta}\right\|_2^2 + 2R_n^2\Delta\boldsymbol{\theta}^{\mathrm{T}}\mathbf{U}^{\mathrm{T}}\Lambda\mathbf{W}(\widehat{\Lambda} - \Lambda)\mathbf{U}\Delta\boldsymbol{\theta} \\
&= (\mathrm{I.1}) + (\mathrm{I.2}) + (\mathrm{I.3}).
\end{aligned}$$

For (I.1), by **(B0)** and **(B1)**(i), with propbability converging to 1, it holds that

$$(\mathrm{I.1}) = \Delta\boldsymbol{\theta}^{\mathrm{T}}\mathbf{U}^{\mathrm{T}}\Lambda\mathbf{W}\Lambda\mathbf{U}\Delta\boldsymbol{\theta}R_n^2 \geq c_1 M^2 R_n^2.$$

For (I.2), by **(B1)**(ii), with propbability converging to 1, it holds that

$$|(\mathrm{I.2})| = O_p(1)R_{e,n}^2\Delta\boldsymbol{\theta}^{\mathrm{T}}\mathbf{U}^{\mathrm{T}}\mathbf{W}\mathbf{U}\Delta\boldsymbol{\theta}R_n^2 = O_p(1)R_{e,n}^2R_n^2M^2 = o_p(1)R_n^2M^2.$$

For (I.3), by **(B1)**(ii), with propbability converging to 1, it holds that

$$|(\mathrm{I.3})| = O_p(1)2R_{e,n}\Delta\boldsymbol{\theta}^{\mathrm{T}}\mathbf{U}^{\mathrm{T}}\mathbf{W}\mathbf{U}\Delta\boldsymbol{\theta}R_n^2 = O_p(1)R_{e,n}R_n^2M^2 = o_p(1)R_n^2M^2.$$

Thus, (I) is dominated by (I.1).

Then we consider (II). It can be deduced that

$$\begin{aligned}
(\mathrm{II}) &= 2R_n\Delta\boldsymbol{\theta}\mathbf{U}^{\mathrm{T}}\left\{\Lambda\mathbf{W}\boldsymbol{\epsilon} + \Lambda\mathbf{W}(\widehat{\Lambda} - \Lambda)\mathbf{U}\boldsymbol{\theta}^* - \Lambda\mathbf{W}\left(\widehat{\mathbf{Y}} - \mathbf{Y}\right)\right\} \\
&\quad + 2R_n\Delta\boldsymbol{\theta}\mathbf{U}^{\mathrm{T}}\left\{\left(\Lambda - \widehat{\Lambda}\right)\mathbf{W}\boldsymbol{\epsilon} + \left(\Lambda - \widehat{\Lambda}\right)\mathbf{W}\left(\widehat{\Lambda} - \Lambda\right)\mathbf{U}\boldsymbol{\theta}^* - \left(\Lambda - \widehat{\Lambda}\right)\mathbf{W}\left(\widehat{\mathbf{Y}} - \mathbf{Y}\right)\right\}.
\end{aligned}$$

By **(B0)**, we have $|R_n\Delta\boldsymbol{\theta}\mathbf{U}^{\mathrm{T}}\Lambda\mathbf{W}\boldsymbol{\epsilon}| = R_nMp^{1/2}n^{-1/2}O_p(1) = R_n^2MO_p(1)$. By **(B0)**and **(B1)**, we have $|R_n\Delta\boldsymbol{\theta}\mathbf{U}^{\mathrm{T}}\Lambda\mathbf{W}\left(\widehat{\Lambda} - \Lambda\right)\mathbf{U}\boldsymbol{\theta}^*| = R_nR_{e,n}\|\boldsymbol{\theta}^*\|_2MO_p(1) = R_n^2MO_p(1)$. Similarly, we can deduced that $|R_n\Delta\boldsymbol{\theta}\mathbf{U}^{\mathrm{T}}\Lambda\mathbf{W}\left(\widehat{\mathbf{Y}} - \mathbf{Y}\right)| = R_nR_{\mu,n}MO_p(1) = R_n^2MO_p(1)$,

$$|R_n\Delta\boldsymbol{\theta}\mathbf{U}^{\mathrm{T}}\left(\widehat{\Lambda} - \Lambda\right)\mathbf{W}\boldsymbol{\epsilon}| = O_p(1)R_nMR_{e,n}\|\mathbf{U}^{\mathrm{T}}\mathbf{W}\boldsymbol{\epsilon}\|_2 = O_p(1)R_nMR_{e,n}\sqrt{\frac{p}{n}} = o_p(1)R_n^2M,$$

$$|R_n\Delta\boldsymbol{\theta}\mathbf{U}^{\mathrm{T}}\left(\widehat{\Lambda} - \Lambda\right)\mathbf{W}\left(\widehat{\Lambda} - \Lambda\right)\mathbf{U}\boldsymbol{\theta}^*| = O_p(1)R_nMR_{e,n}^2\|\boldsymbol{\theta}^*\|_2 = o_p(1)R_n^2M,$$

$$|R_n \Delta\boldsymbol{\theta}\mathbf{U}^{\mathrm{T}}\left(\widehat{\Lambda}-\Lambda\right)\mathbf{W}\left(\widehat{\mathbf{Y}}-\mathbf{Y}\right)| = O_p(1)R_nR_{\mu,n}R_{e,n}M\|\mathbf{W}^{1/2}\mathbf{U}\|_2 = o_p(1)R_n^2M.$$

Thus, (II) is dominated by (I.1) too.

Finally, we consider (III). It can be deduced that

$$\begin{aligned}\text{(III)} &= \sum_{j=1}^{p}\left\{\rho(|\theta_j^* + R_n\Delta\theta_j|;\lambda_1) - \rho(|\theta_j^*|;\lambda_1)\right\} + \sum_{j=p+1}^{2p}\left\{\rho(|\theta_j^* + R_n\Delta\theta_j|;\lambda_1) - \rho(|\theta_j^*|;\lambda_2)\right\}\\ &= \text{(III.1)} + \text{(III.2)}.\end{aligned}$$

It can be seen that

$$\begin{aligned}\text{(III.1)} &\geq \sum_{j\in\mathcal{D}_1}\dot{\rho}(|\alpha_j^*|;\lambda_1)(|\alpha_j^* + R_n\Delta\alpha_j| - |\alpha_j^*|) + \frac{1}{2}\sum_{j\in\mathcal{D}_1}\ddot{\rho}(|\alpha_j^*|;\lambda_1)(|\alpha_j^* + R_n\Delta\alpha_j| - |\alpha_j^*|)^2(1+o(1))\\ &\geq -\sum_{j\in\mathcal{D}_1}\dot{\rho}(|\alpha_j^*|;\lambda_1)R_n|\Delta\alpha_j| - \frac{1}{2}\sum_{j\in\mathcal{D}_1}\ddot{\rho}(|\alpha_j^*|;\lambda_1)R_n^2|\Delta\alpha_j|^2(1+o(1))\\ &\geq -\max_{j\in\mathcal{D}_1}\left\{\dot{\rho}(|\alpha_j^*|;\lambda_1)\right\}\sqrt{d_{1n}}R_nM - \frac{1}{2}\max_{j\in\mathcal{D}_1}\left\{\ddot{\rho}(|\alpha_j^*|;\lambda_1)\right\}R_n^2M^2(1+o(1))\\ &= -O(1)R_n^2M - o(1)R_n^2M^2.\end{aligned}$$

The last equation holds by **(B3)**(i)(ii). Similarly, we can obtain that (III.2) $\geq -O(1)R_n^2M - o(1)R_n^2M^2$. Thus, (III) is dominated by (I.1) too.

Now we can see that all terms are dominated by (I.1), that is, for large enough constant M, $\min_{||\Delta\boldsymbol{\theta}||_2=M}\ell_{\lambda_1,\lambda_2}(\boldsymbol{\theta}^* + R_n\Delta\boldsymbol{\theta},\widehat{\eta}|\mathcal{OB}) > \ell_{\lambda_1,\lambda_2}(\boldsymbol{\theta}^*,\widehat{\eta}|\mathcal{OB})$ holds with probability converging to 1. This completes the proof.

Proof of Theorem 2

For $1 \leq j \leq p$, if $\theta_j = 0$, but $\widehat{\theta}_j \neq 0$, then it holds that

$$0 = -\mathbf{U}_j^{\mathrm{T}}\widehat{\Lambda}\mathbf{W}\left(\widehat{\mathbf{Y}} - \widehat{\Lambda}\mathbf{U}\widehat{\boldsymbol{\theta}}\right) + \dot{\rho}(|\widehat{\theta}_j|) = \text{(I)} + \text{(II)}.$$

It can be deduced that (I) $= O_p(R_n)$. By **(B2)**(ii), (II)$/\lambda_1 > 0$ with probability converging to 1. Thus (II) $> M\lambda_1$, where $M > 0$ is a constant. However, $\lambda_1/R_n \to \infty$, as $n \to \infty$, which comes to the contradictory. For $p+1 \leq j \leq 2p$, if $\theta_j = 0$, but $\widehat{\theta}_j \neq 0$, then it can be similarly deduced the contradictory since $\lambda_2/R_n \to \infty$, as $n \to \infty$.

Proof of Theorem 3

Define the derivative for nuisance function

$$D_*[\Delta\eta] = \partial_t\mathbb{E}\boldsymbol{\phi}\left(\widetilde{\mathbf{Z}},\widetilde{T};\boldsymbol{\theta}_{\mathcal{D}}^*,\eta^* + t\Delta\eta\right)\Big|_{t=0}.$$

Let $\mathbf{J}_* := \partial_{\boldsymbol{\theta}_\mathcal{D}} \mathbb{E}\boldsymbol{\phi}(\widetilde{\mathbf{Z}}, \widetilde{T}; \boldsymbol{\theta}_\mathcal{D}, \eta^*) = \mathbb{E}\left\{(1 - e^*(\mathbf{Z})^2)\mathbf{U}\mathbf{U}^{\mathrm{T}}\right\}$. By Taylor expansion, we have

$$\begin{aligned}
&\mathbb{E}\boldsymbol{\phi}\left(\widetilde{\mathbf{Z}}, \widetilde{T}; \widehat{\boldsymbol{\theta}}_\mathcal{D}, \widehat{\eta}\right) - \mathbb{E}\boldsymbol{\phi}\left(\widetilde{\mathbf{Z}}, \widetilde{T}; \boldsymbol{\theta}^*_\mathcal{D}, \eta^*\right) \\
&= \mathbf{J}_*\left(\widehat{\boldsymbol{\theta}}_\mathcal{D} - \boldsymbol{\theta}^*_\mathcal{D}\right) + D_*[\widehat{\eta} - \eta^*] + \partial_t^2 \mathbb{E}\boldsymbol{\phi}\left(\widetilde{\mathbf{Z}}, \widetilde{T}; \boldsymbol{\theta}^*_\mathcal{D} + t\left(\widehat{\boldsymbol{\theta}}_\mathcal{D} - \boldsymbol{\theta}^*_\mathcal{D}\right), \eta^* + t\left(\widehat{\eta} - \eta^*\right)\right)\Big|_{t=\bar{t}}.
\end{aligned}$$

For any measurable function f, we use the notation $\widetilde{\mathbb{E}}_n f(\widetilde{\mathbf{Z}}, \widetilde{T}) = \sum_{i=1}^n w_i f(\widetilde{\mathbf{Z}}_i, \widetilde{T}_i)$ for empirical expectation under Stute's measure. Similarly, we use the notation $\widetilde{\mathbb{G}}_n f(\widetilde{\mathbf{Z}}, \widetilde{T}) = \sqrt{n}(\sum_{i=1}^n w_i f(\widetilde{\mathbf{Z}}_i, \widetilde{T}_i) - \mathbb{E}f(\widetilde{\mathbf{Z}}, \widetilde{T}))$ for empirical process under Stute's measure. For our model, we have

$$\widetilde{\mathbb{E}}_n \boldsymbol{\phi}\left(\widetilde{\mathbf{Z}}, \widetilde{T}; \widehat{\boldsymbol{\theta}}_\mathcal{G}, \widehat{\eta}\right) + \partial_{\boldsymbol{\theta}_\mathcal{D}} \rho\left(\widehat{\boldsymbol{\theta}}_\mathcal{D}\right) = \mathbf{0},$$

where $\widetilde{\mathbb{E}}_n \boldsymbol{\phi}(\widetilde{\mathbf{Z}}, \widetilde{T}; \boldsymbol{\theta}_\mathcal{D}, \eta) = -\mathbf{U}^{\mathrm{T}}\Lambda\mathbf{W}\mathbf{Y} + \mathbf{U}^{\mathrm{T}}\Lambda\mathbf{W}\Lambda\mathbf{U}\boldsymbol{\theta}_\mathcal{D}$. By this equation and the result of Taylor expansion, it holds that

$$\begin{aligned}
\sqrt{n}\mathbf{q}^{\mathrm{T}}\mathbf{J}_*\left(\widehat{\boldsymbol{\theta}}_\mathcal{D} - \boldsymbol{\theta}^*_\mathcal{D}\right) &= \sqrt{n}\mathbf{q}^{\mathrm{T}}\widetilde{\mathbb{E}}_n\boldsymbol{\phi}\left(\widetilde{\mathbf{Z}}, \widetilde{T}; \boldsymbol{\theta}^*_\mathcal{D}, \eta^*\right) - \sqrt{n}\mathbf{q}^{\mathrm{T}}D_*\left[\widehat{\eta} - \eta^*\right] \\
&\quad - \sqrt{n}\mathbf{q}^{\mathrm{T}}\partial_t^2\mathbb{E}\boldsymbol{\phi}\left(\widetilde{\mathbf{Z}}, \widetilde{T}; \boldsymbol{\theta}^*_\mathcal{D} + t\left(\widehat{\boldsymbol{\theta}}_\mathcal{D} - \boldsymbol{\theta}^*_\mathcal{D}\right), \eta^* + t\left(\widehat{\eta} - \eta^*\right)\right)\Big|_{t=\bar{t}} \\
&\quad + \mathbf{q}^{\mathrm{T}}\widetilde{\mathbb{G}}_n\left\{\boldsymbol{\phi}\left(\widetilde{\mathbf{Z}}, \widetilde{T}; \widehat{\boldsymbol{\theta}}_\mathcal{D}, \widehat{\eta}\right) - \boldsymbol{\phi}\left(\widetilde{\mathbf{Z}}, \widetilde{T}; \boldsymbol{\theta}^*_\mathcal{D}, \eta^*\right)\right\} \\
&\quad + \sqrt{n}\mathbf{q}^{\mathrm{T}}\partial_{\boldsymbol{\theta}_\mathcal{D}}\rho\left(\widehat{\boldsymbol{\theta}}_\mathcal{D}\right) \\
&= \text{(I)} + \text{(II)} + \text{(III)} + \text{(IV)},
\end{aligned}$$

where $\bar{t} \in (0, 1)$.

First, we consider (I). It can be deduced that $D_*[\widehat{\eta} - \eta^*] = \mathbf{0}$. Then by **(B0)** and σ_*^2 defined in the conditions, we have (I) $\to_d N(0, \sigma_*^2)$. Then for (II), it can be deduced that

$$\|\partial_t^2\mathbb{E}\boldsymbol{\phi}\left(\widetilde{\mathbf{Z}}, \widetilde{T}; \boldsymbol{\theta}^*_\mathcal{D} + t\left(\widehat{\boldsymbol{\theta}}_\mathcal{D} - \boldsymbol{\theta}^*_\mathcal{D}\right), \eta^* + t\left(\widehat{\eta} - \eta^*\right)\right)|_{t=\bar{t}}\|_2 = O(1)R_n^2.$$

Then by **(B3)**(iv), we have $|\text{(II)}| \leq \sqrt{n}R_n^2 O(1) = o(1)$. For (IV), we have

$$\begin{aligned}
|\text{(IV)}| &\leq \sqrt{n}\|\partial_{\boldsymbol{\theta}_\mathcal{D}}\rho(\boldsymbol{\theta}^*_\mathcal{D})\|_2 + \sqrt{n}\|\partial_{\boldsymbol{\theta}_\mathcal{D}}\rho(\widehat{\boldsymbol{\theta}}_\mathcal{D}) - \partial_{\boldsymbol{\theta}_\mathcal{D}}\rho(\boldsymbol{\theta}^*_\mathcal{D})\|_2 \\
&\leq \sqrt{nd_{1n}\left(\max_{j\in\mathcal{D}_1}\left\{\dot{\rho}(|\theta_j^*|; \lambda_1)\right\}\right)^2 + nd_{2n}\left(\max_{j\in\mathcal{D}_2}\left\{\dot{\rho}(|\theta_j^*|; \lambda_2)\right\}\right)^2} \\
&\quad + O_p(1)\sqrt{n}\max\left\{\lambda_1, \lambda_2\right\}R_n
\end{aligned}$$

By **(B3)**(iii)(iv), it follows that $|\text{(IV)}| = o(1)$. Now we consider (III). Define class

$$\mathcal{F}_2 = \left\{\phi_j\left(\cdot; \boldsymbol{\theta}_\mathcal{D}, \widehat{\eta}\right) - \phi_j\left(\cdot; \boldsymbol{\theta}^*_\mathcal{D}, \eta^*\right) : j \in \mathcal{D}, \|\boldsymbol{\theta}_\mathcal{D} - \boldsymbol{\theta}^*_\mathcal{D}\|_2 \leq cR_n\right\},$$

where c is some positve constant. Then we can see that

$$|(\mathrm{III})| \le \sqrt{d_n} \sup_{f\in\mathcal{F}_2} |\widetilde{\mathbb{G}}_n f|.$$

By **(B3)**(v), we have $\sup_{f\in\mathcal{F}_2} \|f\|^2_{F^0,2} \le O(1)R_n^b$. Consider that

$$\sup_{f\in\mathcal{F}_2} |f| \le \sup_{f\in\mathcal{F}_{1,\widehat{\eta}}} |f| + \sup_{f\in\mathcal{F}_{1,\eta^*}} |f| \le F_{1,\widehat{\eta}} + F_{1,\eta^*},$$

the function $F_2 := F_{1,\widehat{\eta}} + F_{1,\eta^*}$ can be the envelop for $\mathcal{F}_2$. Since $\mathcal{F}_2 \subset \mathcal{F}_{1,\widehat{\eta}} - \mathcal{F}_{1,\eta^*}$, by **(B4)**, it holds that

$$\begin{aligned}
&\sup_Q \log N(\xi\|F_2\|_{Q,2}, \mathcal{F}_2, \|\cdot\|_{Q,2})\\
&\le \sup_Q \log N\left(\frac{\xi}{2}\|F_{1,\widehat{\eta}}\|_{Q,2}, \mathcal{F}_{1,\widehat{\eta}}, \|\cdot\|_{Q,2}\right) + \sup_Q \log N\left(\frac{\xi}{2}\|F_{1,\eta^*}\|_{Q,2}, \mathcal{F}_{1,\eta^*}, \|\cdot\|_{Q,2}\right)\\
&\le 2v\log\frac{2c_6}{\xi}.
\end{aligned}$$

Under the condition **(B0)**, **(B3)**(v), and by Lemma 6.2 in Chernozhukov et al (2018), with probability converging to 1, for $q > 2$, it holds that $\sup_{f\in\mathcal{F}_2} |\widetilde{\mathbb{G}}_n f| \le O\left(R_n^{b/2} + n^{-1/2+1/q}\right)$. Thus by **(B3)**(v), we have (III) $\le \sqrt{d_n} O(R_n^{b/2} + n^{-1/2+1/q}) = o(1)$.

Finally, combining the analysis of (I) to (IV), we have $\sqrt{n}\mathbf{q}^{\mathrm{T}}\mathbf{J}_*(\widehat{\boldsymbol{\theta}}_{\mathcal{D}} - \boldsymbol{\theta}^*_{\mathcal{D}}) \to_d N(0, \sigma^2_*)$. This completes the proof.

Proof of Theorem 5

For abuse of notation, here we still use $\mathbf{X}$ to denote $(A - e(\mathbf{Z}))\mathbf{X}$. Let

$$\begin{aligned}
\mathbf{V}_{rct} &= \mathbb{E}\left(S\mathbf{X}_{\mathcal{D}_1}\mathbf{X}^{\mathrm{T}}_{\mathcal{D}_1}\right),\\
\mathbf{V}_{int} &= \left[\mathbb{E}\mathbf{X}_{\mathcal{D}_1}\mathbf{X}^{\mathrm{T}}_{\mathcal{D}_1} - \left\{\mathbb{E}(1-S)\mathbf{X}_{\mathcal{D}_1}\mathbf{X}^{\mathrm{T}}_{\mathcal{D}_2}\right\}\left\{\mathbb{E}(1-S)\mathbf{X}_{\mathcal{D}_2}\mathbf{X}^{\mathrm{T}}_{\mathcal{D}_2}\right\}^{-1}\left\{\mathbb{E}(1-S)\mathbf{X}_{\mathcal{D}_2}\mathbf{X}^{\mathrm{T}}_{\mathcal{D}_1}\right\}\right].
\end{aligned}$$

When there is no censoring, let $\mathbf{q} \in \mathbb{R}^{d_{1n}}$, it can be seen that as $n \to \infty$,

$$\begin{aligned}
\mathrm{Var}(\sqrt{n}\mathbf{q}^{\mathrm{T}}\widehat{\boldsymbol{\alpha}}^{rct}_{\mathcal{D}_1}) &\to \frac{1}{r_S}\mathbf{q}^{\mathrm{T}}\left\{\mathbb{E}\left(\mathbf{X}_{\mathcal{D}_1}\mathbf{X}^{\mathrm{T}}_{\mathcal{D}_1}|S=1\right)\right\}^{-1}\mathbf{q}^{\mathrm{T}} = \mathbf{q}^{\mathrm{T}}\mathbf{V}^{-1}_{rct}\mathbf{q}^{\mathrm{T}},\\
\mathrm{Var}(\sqrt{n}\mathbf{q}^{\mathrm{T}}\widehat{\boldsymbol{\alpha}}_{\mathcal{D}_1}) &\to \mathbf{q}^{\mathrm{T}}\mathbf{V}^{-1}_{int}\mathbf{q}^{\mathrm{T}}.
\end{aligned}$$

It can be deduced that

$$
\begin{aligned}
&\mathbf{V}_{rct} - \mathbf{V}_{int} \\
&= \mathbb{E}(1-S)\mathbf{X}_{\mathcal{D}_1}\mathbf{X}_{\mathcal{D}_1}^{\mathrm{T}} - \left\{\mathbb{E}(1-S)\mathbf{X}_{\mathcal{D}_1}\mathbf{X}_{\mathcal{D}_2}^{\mathrm{T}}\right\}\left\{\mathbb{E}(1-S)\mathbf{X}_{\mathcal{D}_2}\mathbf{X}_{\mathcal{D}_2}^{\mathrm{T}}\right\}^{-1}\left\{\mathbb{E}(1-S)\mathbf{X}_{\mathcal{D}_2}\mathbf{X}_{\mathcal{D}_1}^{\mathrm{T}}\right\}.
\end{aligned}
$$

By the theory of projection, $\mathbf{V}_{rct} - \mathbf{V}_{int} \geq 0$, where the equality holds if and only if there exists a $d_{2n} \times d_{1n}$ constant matrix $\mathbf{Q}$, such that when $S = 0$, it holds that $\mathbf{X}_{\mathcal{D}_1} = \mathbf{Q}^{\mathrm{T}}\mathbf{X}_{\mathcal{D}_2}$.

As a result,

$$
\mathrm{Var}(\sqrt{n}\mathbf{q}^{\mathrm{T}}\widehat{\boldsymbol{\alpha}}_{\mathcal{D}_1})^{-1} - \mathrm{Var}(\sqrt{n}\mathbf{q}^{\mathrm{T}}\widehat{\boldsymbol{\alpha}}_{\mathcal{D}_1}^{rct})^{-1} \geq 0, \tag{1}
$$

where the equality holds if and only if there exists a $d_{2n} \times d_{1n}$ constant matrix $\mathbf{Q}$, such that when $S = 0$, it holds that $\mathbf{X}_{\mathcal{D}_1} = \mathbf{Q}^{\mathrm{T}}\mathbf{X}_{\mathcal{D}_2}$. Specially, when $\mathcal{D}_1 \subset \mathcal{D}_2$, the equality holds. When $\mathbf{X}_{\mathcal{D}_1} \perp \mathbf{X}_{\mathcal{D}_2} | S = 0$ or $\mathcal{D}_2 = \emptyset$, under (B1)(i), it holds that $\mathbf{V}_{rct} - \mathbf{V}_{int} = \mathbb{E}(1-S)\mathbf{X}_{\mathcal{D}_1}\mathbf{X}_{\mathcal{D}_1}^{\mathrm{T}} > 0$, which means the inequality in (1) strictly holds.

Additional assumptions for Theorem 4

We need some conditions similar to **(B0)-(B4)** to derive the asymptotic properties of the RCT-only estimator. Denote that the true parameters for HTE be $\boldsymbol{\alpha}^*$, and true nuisance functions be η^*. We first introduce some notations. Following the notations in Stute (1996), let G_r be the probability distribution function (p.d.f) of C conditional on $S = 1$, with $\tau_{Gr} = \inf\{x : G_r(x) = 1\}$, F_r be the p.d.f of $\widetilde{T}$ conditional on $S = 1$, with $\tau_{Fr} = \inf\{x : F_r(x) = 1\}$, and H_r be the p.d.f of T conditional on $S = 1$, with $\tau_{H_r} = \inf\{x : H_r(x) = 1\}$. Let F_r^0 be the p.d.f of $(\widetilde{\mathbf{X}}, \widetilde{T})$ conditional on $S = 1$ with $\widetilde{\mathbf{X}} = (\mathbf{X}, A)$. Define

$$
\widetilde{F}_r^0(\mathbf{x}, t) = \begin{cases} F_r^0(\mathbf{x}, t), & t < \tau_{H_r}, \\ F_r^0(\mathbf{x}, \tau_{H_r}-) + F_r^0(\mathbf{x}, \tau_{H_r}) I(\tau_{H_r} \in \mathcal{H}_r), & t \geq \tau_{H_r}, \end{cases}
$$

with $\mathcal{H}_r$ denoting the set of atoms of H_r, possibly empty. Define the score function

$$
\varphi_j(\widetilde{\mathbf{X}}, \widetilde{T}; \boldsymbol{\alpha}^*, \eta^*) = \{A - e^*(\mathbf{X}, 1)\}\mathbf{X}_j\left[\log\widetilde{T} - \mu^*(\mathbf{X}, 1) - \{A - e^*(\mathbf{X}, 1)\}\mathbf{X}^{\mathrm{T}}\boldsymbol{\alpha}^*\right], \quad j = 1, 2, ..., p.
$$

It can be seen that $\mathbb{E}\boldsymbol{\varphi}(\widetilde{\mathbf{X}}, \widetilde{T}; \boldsymbol{\alpha}^*, \eta^*) = \mathbf{0}$. Define $\widetilde{H}_r^1(\mathbf{x}, y) = \Pr(\widetilde{\mathbf{X}} \leq \mathbf{x}, T \leq y, \delta = 1 | S = 1)$, $\widetilde{H}_r^0(y) = \Pr(T \leq y, \delta = 0 | S = 1)$, and

$$
\gamma_{r0}(y) = \exp\left\{\int_0^{y-} \frac{\widetilde{H}_r^0(dv)}{1 - H_r(v)}\right\},
$$

$$
\gamma_{r1j}(y) = \frac{1}{1 - H_r(y)}\int 1_{\{y<v\}}\varphi_j(\mathbf{z}, v; \boldsymbol{\alpha}^*, \eta^*)\gamma_{r0}(v)\widetilde{H}_r^1(d\mathbf{z}, dv), \quad j = 1, 2, ..., p,
$$

$$\gamma_{r2j}(y) = \int\int \frac{1_{\{v<y,v<w\}}\varphi_j(\mathbf{z}, w; \boldsymbol{\alpha}^*, \eta^*)\gamma_{r0}(v)}{\{1 - H_r(v)\}^2} \widetilde{H}_r^0(dv)\widetilde{H}_r^1(d\mathbf{z}, dw), \quad j = 1, 2, ..., p.$$

Let $\boldsymbol{\gamma}_{r1}(y) = \{\gamma_{r1j}(y), j = 1, 2, ..., p\}$, and $\boldsymbol{\gamma}_{r2}(y) = \{\gamma_{r2j}(y), j = 1, 2, ..., p\}$.

(C0) For $j = 1, 2, ..., 2p$,

(i) $\Pr(\widetilde{T} \leq C|\mathbf{X}, S = 1, A, \widetilde{T}) = \Pr(\widetilde{T} \leq C|S = 1, \widetilde{T})$.

(ii) The p.d.f. F_r and G_r have no jump in common, and $\tau_{Fr} < \tau_{Gr}$.

(iii) $\mathbb{E}\left\{\varphi_j(\widetilde{\mathbf{Z}}, T; \boldsymbol{\alpha}^*, \eta^*)\gamma_{r0}(T)\delta|S = 1\right\}^2 < \infty$.

(iv) Let $g_r(y) = \int_0^{y-} \{1 - H_r(w)\}^{-1}\{1 - G_r(w)\}^{-1} G_r(dw)$. It holds that

$$\int |\varphi_j(\mathbf{z}, w; \boldsymbol{\alpha}^*, \eta^*)|\sqrt{g_r(w)}\widetilde{F}_r^0(d\mathbf{z}, dw) < \infty.$$

(C1) (i) The eigenvalues of $\mathbb{E}\left[\{A - e(\mathbf{X}, 1)\}^2 \mathbf{X}\mathbf{X}^{\mathrm{T}}|S = 1\right]$ are larger than a positive constant c_{r1}.

(ii) The eigenvalues of $\mathbb{E}\left(\mathbf{X}\mathbf{X}^{\mathrm{T}}|S = 1\right)$ are smaller than a positive constant c_{r2}.

(C2) Let $R_{e,n}$ be the convergence rate of $\|\widehat{e} - e^*\|_\infty$, $R_{\mu,n}$ be the convergence rate of $\|\widehat{\mu} - \mu^*\|_\infty$. Define rate $R_{n_1} = \max\left\{R_{\mu,n}, R_{e,n}\|\boldsymbol{\theta}^*\|_2, \sqrt{p/n}\right\}$. Let $\mathcal{D}_1 = \left\{j|\alpha_j^* \neq 0\right\}$ with element number d_{1n}. Define $\dot{\rho}(x; \lambda) = \partial\rho(x; \lambda)/\partial x$, $\ddot{\rho}(x; \lambda) = \partial^2\rho(x; \lambda)/\partial x^2$, for $x > 0$.

(i) $\sqrt{d_{1n}} \max\left\{|\dot{\rho}(|\alpha_j^*|; \lambda_1)|, j = 1, 2, ..., p\right\} = O_p(n_1^{-1/2})$.

(ii) $\sqrt{n_1}\lambda_1 R_{n_1} = o(1)$.

(iii) $\mathbb{E}|\varphi_j(\widetilde{\mathbf{X}}, \widetilde{T}; \boldsymbol{\alpha}, \eta) - \varphi_j(\widetilde{\mathbf{X}}, \widetilde{T}; \boldsymbol{\alpha}^*, \eta^*)|^2 \leq (\|\boldsymbol{\alpha} - \boldsymbol{\alpha}^*\|_2 \wedge \|\eta - \eta^*\|_\infty)^{b_r} c_{r3}$, where c_{r3} is some positive constant. In addition, $\sqrt{d_{1n}}R_{n_1}^{b_r/2} = o(1)$, $\sqrt{d_{1n}}n_1^{-1/2+1/q} = o(1)$, $q > 2$.

(C3) Let $\Theta_r = \{\boldsymbol{\alpha} : \|\boldsymbol{\alpha} - \boldsymbol{\alpha}^*\|_2 \leq R_{n_1}c_{r4}\}$, where c_{r4} is some positive constant. Define class

$$\mathcal{F}_{r1,\eta} = \{\varphi_j(\cdot; \boldsymbol{\alpha}, \eta) : j \in \mathcal{D}_1, \boldsymbol{\alpha} \in \Theta_r\},$$

with measurable envelop $F_{r1,\eta}$. It satisfies $\|F_{r1,\eta}\|_{F_r^0,q} \leq c_{r5}$ where c_{r5} is some positive constant. It holds that for all $0 < \xi \leq 1$,

$$\sup_Q \log N(\xi\|F_{r1,\eta}\|_{Q,2}, \mathcal{F}_{r1,\eta}, \|\cdot\|_{Q,2}) \leq v \log \frac{c_{r6}}{\xi},$$

where c_{r6} is some positive constant.

Additional simulation experiments

1. **Weaker signal of the coefficients**: let $Signal = 1$, and the other model settings remain the same as in Section 4 in the paper. The simulation results based on 500 replicates are shown in Table 1 and Table 2. The proposed method is still eective. Compared with the results of $Signal = 2$, it can be observed that when the signal of parameters get stronger, for all methods, RMSE, TIR, FDR improve.

2. **Logistic distribution of the error term**: let the error term follow the Logistic distribution instead of the Normal distribution, i.e., the survival time follows the log-Logistic distribution. We consider the case where the dimension of covariates is $p = 20$; the censoring rate is CR= 40% and $Signal = 2$ for the proposed model and outcome adjusted model. The other model settings remain the same asin Section 4 of the paper. The simulation results based on 500 replicates are shown in Table 3. It can be seen that the proposed method still performs well and is more efficient than the outcome-adjusted method. Compared with the log-Normal distribution, all the methods lose some efficiency, i.e., higher RMSE.

3. **Severe censoring**: Let the censoring rate be CR= 60%. We consider the case where the dimension of covariates is $p = 20$ and $Signal = 2$ for the proposed model and outcome-adjusted model. The other model settings remain the same as in Section 4 of the paper. The simulation results based on 500 replicates are shown in Table 4. It can be seen that the proposed method still works well and is more ecient than the outcome-adjusted method.

Table 1: The RMSE ($\times 10^2$) of the HTE estimation when $Signal = 1$ over 500 experiment replicates

Methods	with unmeasured confounding				no unmeasured confounding			
	p=20		p=50		p=20		p=50	
	CR=20%	CR=40%	CR=20%	CR=40%	CR=20%	CR=40%	CR=20%	CR=40%
**RL.or**	6.54	7.52	4.15	4.97	4.59	5.37	2.96	3.42
**RL.RCTor**	10.10	11.87	6.22	7.45	10.10	11.87	6.22	7.45
**RL.NAIor**	35.75	35.96	22.54	22.72				
**RL.cv**	**7.06**	**9.05**	**5.41**	**9.32**	**4.91**	**5.68**	**3.08**	**3.53**
**RL.bic**	10.56	16.94	12.60	17.55	4.68	5.47	2.99	3.46
**RL.RCT**	11.08	14.29	7.03	9.52	11.08	14.29	7.03	9.52
**RL.NAI**	37.05	37.78	23.92	24.45				
**OA.or**	7.30	8.41	4.70	5.67	5.60	6.70	3.68	4.48
**OA.RCTor**	12.43	14.60	8.70	10.18	12.43	14.60	8.70	10.18
**OA.cv**	**7.69**	**9.67**	**6.27**	**9.44**	**5.73**	**6.85**	**3.73**	**4.56**
**OA.bic**	11.94	17.22	13.25	16.93	5.63	6.73	3.70	4.50
**OA.RCT**	12.65	15.09	8.92	10.68	12.65	15.09	8.92	10.68
**GM0.or**	8.71	10.18	5.82	6.96	6.46	7.50	4.18	4.98
**GM0.RCTor**	13.97	16.39	9.64	11.47	13.97	16.39	9.62	11.48
**GM0.cv**	**9.29**	**11.64**	**7.04**	**9.99**	**7.03**	**8.37**	**4.54**	**5.47**
**GM0.biv**	10.01	14.90	10.48	14.61	6.65	7.90	4.30	5.12
**GM0.RCT**	14.33	17.55	10.61	13.99	14.33	17.55	10.56	14.00
**GM1.or**	7.91	9.26	5.29	6.34	6.11	7.22	3.97	4.77
**GM1.RCTor**	13.17	15.34	9.12	10.49	13.18	15.29	9.12	10.49
**GM1.cv**	**8.53**	**10.69**	**6.22**	**9.20**	**6.53**	**7.85**	**4.26**	**5.17**
**GM1.bic**	9.03	13.42	9.33	14.27	6.34	7.42	4.07	4.85
**GM1.RCT**	13.48	16.11	9.74	12.28	13.49	16.05	9.72	12.23
**Meta.or**	7.44	8.72	5.00	5.98	5.92	6.93	3.84	4.58
**Meta.RCTor**	12.64	14.70	8.80	10.17	12.65	14.68	8.79	10.17
**Meta.cv**	**7.81**	**9.76**	**5.82**	**8.33**	**6.21**	**7.38**	**4.06**	**4.89**
**Meta.bic**	8.35	12.39	8.65	12.72	6.07	7.13	3.92	4.66
**Meta.RCT**	12.89	15.46	9.49	12.05	12.90	15.44	9.48	12.04
**GM01.or**	8.55	10.37	5.93	7.39	6.60	8.21	4.29	5.51
**GM01.RCTor**	15.10	19.23	10.56	13.53	15.09	19.26	10.53	13.52
**GM01.cv**	**9.55**	**12.21**	**7.03**	**9.87**	**8.06**	**10.63**	**5.45**	**8.35**
**GM01.bic**	9.10	11.50	6.51	8.72	7.64	9.86	5.00	7.18
**GM01.RCT**	15.39	19.71	10.74	14.14	15.39	19.73	10.71	14.11

Some results are marked in bold to make it clear for readers to compare different methods. The results that behave the best, except for the Oracle estimates (i.e., the smallest RMSE), are marked with underlines.

In the table, CR represents cencoring rate. Among these methods, those with names starting with “RL" indicate the proposed model. RL.cv and RL.bic represent the proposed estimates under CV and BIC criteria, respectively. RL.RCT represents the estimate merely based on RCT data. RL.NAI is the naive estimate that completely ignores unmeasured confounding effect. RL.or, RL.RCTor, and RL.NAIor are the oracle estimates of the integrative, RCT-only, and naive analysis, respectively. Other methods with names starting with “OA”, “GM0”, “GM1”, “Meta” and “GM01” are introduced in detail in Section 4.

Table 2: The averaged TIR(/%) and FDR(/%) when $Signal = 1$ over 500 experiment replicates.

Index	Methods	with unmeasured confounding				no unmeasured confounding			
		p=20		p=50		p=20		p=50	
		CR=20%	CR=40%	CR=20%	CR=40%	CR=20%	CR=40%	CR=20%	CR=40%
TIR	**RL.cv**	100	100	100	100	93.00	92.80	94.00	90.80
	**RL.bic**	100	100	100	99.00	97.40	96.40	95.40	94.40
	**OA.cv**	100	100	100	100	96.40	93.20	95.60	94.00
	**OA.bic**	100	100	100	99.00	98.00	97.60	97.60	95.80
	**GM0.cv**	100	100	100	99.73	83.60	81.20	81.61	77.95
	**GM0.biv**	100	100	100	99.47	89.20	88.20	92.41	88.98
	**GM1.cv**	100	100	100	100	85.20	80.60	82.53	77.95
	**GM1.bic**	100	100	100	100	91.00	86.20	88.97	86.35
	**Meta.cv**	100	100	100	100	73.20	67.40	70.57	63.78
	**Meta.bic**	100	100	100	100	83.40	77.20	82.76	78.74
	**GM01.cv**	100	100	100	100	17.20	6.20	9.20	0.26
	**GM01.bic**	100	100	100	100	32.40	14.20	19.77	3.15
FDR	**RL.cv**	2.49	5.62	6.59	11.87	1.62	1.58	1.60	1.58
	**RL.bic**	1.25	2.87	3.06	3.98	0.35	0.28	0.36	0.52
	**RL.RCT**	6.39	11.16	8.38	15.55	6.39	11.16	8.38	15.55
	**RL.NAI**	5.29	5.65	4.41	4.61				
	**OA.cv**	2.16	4.96	5.19	8.40	0.94	1.20	1.00	1.65
	**OA.bic**	1.34	2.11	1.60	2.41	0.13	0.13	0.35	0.31
	**OA.RCT**	2.88	5.10	3.82	7.72	2.88	5.10	3.82	7.72
	**GM0.cv**	5.04	8.56	8.33	15.75	3.76	4.90	4.62	7.29
	**GM0.biv**	2.32	3.47	4.04	6.57	0.90	1.32	0.63	1.69
	**GM0.RCT**	2.88	5.02	2.57	6.15	2.88	5.02	2.54	6.09
	**GM1.cv**	3.86	8.11	7.35	15.23	3.00	4.28	4.47	6.29
	**GM1.bic**	2.08	3.35	3.96	6.22	0.90	0.80	0.89	1.25
	**GM1.RCT**	2.88	4.17	2.23	4.48	2.90	4.24	2.34	4.82
	**Meta.cv**	7.72	13.72	13.18	25.33	5.71	7.80	8.08	11.38
	**Meta.bic**	3.87	5.95	7.02	11.14	1.64	2.01	1.39	2.71
	**Meta.RCT**	5.51	8.38	4.61	9.92	5.48	8.42	4.64	10.18
	**GM01.cv**	7.84	16.61	20.29	41.2	6.53	15.23	15.73	36.5
	**GM01.bic**	4.36	9.37	11.26	24.60	4.10	9.11	9.46	24.07
	**GM01.RCT**	5.34	9.01	3.81	9.09	5.49	8.81	3.74	8.56

In the table, CR represents the cencoring rate. TIR is the rate of correctly identifying the real case where unmeasured confounding effect exists or not. FDR is the false discovery rate of the HTE estimates. Among these methods, those with names starting with "RL" indicate the proposed model. RL.cv and RL.bic represent the proposed estimates under CV and BIC criteria, respectively. RL.RCT represents the estimate merely based on RCT data. RL.NAI is the naive estimate that completely ignores unmeasured confounding effect. Other methods with names starting with "OA", "GM0", "GM1", "Meta" and "GM01" are introduced in detail in Section 4.

Table 3: Simulation results of the proposed method (RL) and the outcome-adjusted method (OA) when the survival time follows log-Logistic distribution, CR=40%, $p = 20$.

Index	Method	$\boldsymbol{\beta}^* \neq \mathbf{0}$	$\boldsymbol{\beta}^* = \mathbf{0}$	Index	Method	$\boldsymbol{\beta}^* \neq \mathbf{0}$	$\boldsymbol{\beta}^* = \mathbf{0}$
RMSE$\times 10^2$	RL.or	14.81	10.41	FDR/%	RL.cv	5.24	1.65
	RL.RCTor	22.56	22.56		RL.bic	3.43	0.37
	RL.NAIor	71.86	-		RL.RCT	8.90	8.90
	RL.cv	17.74	11.09		RL.NAI	6.69	-
	RL.bic	27.01	10.55		OA.cv	4.02	1.39
	RL.RCT	25.27	25.27		OA.bic	1.95	0.33
	RL.NAI	75.16	-		OA.RCT	4.51	4.50
	OA.or	16.29	13.19	TIR/%	RL.cv	100.00	91.00
	OA.RCTor	28.91	28.91		RL.bic	100.00	93.40
	OA.cv	18.03	13.43		OA.cv	100.00	93.81
	OA.bic	28.97	13.24		OA.bic	100.00	95.58
	OA.RCT	29.63	29.63				

In the table, the results are based on 500 replicates. CR represents the cencoring rate. $\boldsymbol{\beta}^* \neq \mathbf{0}$ means there is unmeasured confounding effect, otherwise, there is no unmeasured confounding effect. TIR is the rate of correctly identifying the real case where unmeasured confounding effect exists or not. FDR is the false discovery rate of the HTE estimates. RL.cv and RL.bic represent the proposed estimates under the CV and BIC criteria, respectively. RL.RCT represents the estimate merely based on RCT data. RL.NAI is the naive estimate that completely ignores unmeasured confounding effect.

Table 4: Simulation results of the proposed method (RL) and the outcome-adjusted method (OA) when CR=60%, $p = 20$.

Index	Method	$\boldsymbol{\beta}^* \neq \mathbf{0}$	$\boldsymbol{\beta}^* = \mathbf{0}$	Index	Method	$\boldsymbol{\beta}^* \neq \mathbf{0}$	$\boldsymbol{\beta}^* = \mathbf{0}$
RMSE$\times 10^2$	RL.or	14.81	9.13	FDR/%	RL.cv	3.60	1.49
	RL.RCTor	24.20	24.20		RL.bic	2.28	0.44
	RL.NAIor	71.46	-		RL.RCT	9.75	9.75
	RL.cv	16.80	9.74		RL.NAI	6.31	-
	RL.bic	20.77	9.33		OA.cv	1.31	0.53
	RL.RCT	27.71	27.71		OA.bic	0.54	0.19
	RL.NAI	74.23	-		OA.RCT	3.45	3.45
	OA.or	17.08	14.27	TIR/%	RL.cv	100.00	94.80
	OA.RCTor	32.36	32.36		RL.bic	100.00	95.20
	OA.cv	17.56	14.38		OA.cv	100.00	97.74
	OA.bic	18.69	14.31		OA.bic	100.00	98.02
	OA.RCT	32.95	32.95				

In the table, the results are based on 500 replicates. CR represents the cencoring rate. $\boldsymbol{\beta}^* \neq \mathbf{0}$ means there is unmeasured confounding effect, otherwise, there is no unmeasured confounding effect. TIR is the rate of correctly identifying the real case where unmeasured confounding effect exists or not. FDR is the false discovery rate of the HTE estimates. RL.cv and RL.bic represent the proposed estimates under the CV and BIC criteria, respectively. RL.RCT represents the estimate merely based on RCT data. RL.NAI is the naive estimate that completely ignores unmeasured confounding effect.
